# Injury incidence, burden and associated risk factors in walking football players

**DOI:** 10.5114/biolsport.2026.154946

**Published:** 2025-10-31

**Authors:** Maite Lejonagoitia-Garmendia, Iraia Bidaurrazaga-Letona, Iratxe Duñabeitia, Izaro Esain, Rakel Berriozabalgoitia, Begoña Sanz, Xabier Monasterio, Jon Larruskain, Susana M Gil

**Affiliations:** 1 Department of Physiology, Faculty of Medicine and Nursing, University of the Basque Country (EHU), Leioa, Spain; 2 Medical Services, Athletic Club, Lezama, Spain; 3 Biocruces Bizkaia Health Research Institute, Barakaldo, Spain

**Keywords:** Walking football, Injuries, Older adults, Risk factors

## Abstract

Walking football (WF) is an effective activity for older adults to enhance cardiovascular, metabolic and psychosocial health. However, evidence on injury risks remains limited. This study aimed to describe the incidence and burden of injuries during a WF season and identify potential risk factors, including age, muscle function and blood biomarkers. Sixty-four male recreational WF players aged > 50 years participated. Baseline assessments included anthropometry, muscle strength and blood tests. A prospective follow-up recorded injuries and exposure during training (twice weekly, 1 h) and occasional matches. A total of 2,946 player-sessions were documented. Sixty-two injuries occurred (80.6% during training), with an overall incidence of 19.7 injuries per 1000 hours (95% CI: 15.3–25.2) and 892.4 days of injury burden. The most frequent were muscle injuries (64.5%), mainly in the hamstrings (29%) and calf muscles (25.8%). Most were acute (90.3%), non-contact (85.5%), and classified as moderate or severe. Older participants experienced a greater burden of injuries. Muscle functionality was not associated with muscle injury incidence. In contrast, higher incidence (p < 0.05) was observed among players with low HDL cholesterol, elevated triglycerides, high atherogenic index and elevated glucose. Despite the adapted rules, walking football in older adults is associated with an elevated risk of muscle injuries, particularly in the posterior leg compartment. Older age and adverse metabolic profiles appear to be important contributors. Future research should focus on evaluating the balance between health benefits and injury risks, optimizing warm-up routines and training load, and integrating screening for injuryrelated risk factors to enhance player safety.

## INTRODUCTION

Football is the most popular sport worldwide. A form of football adapted for older adults, known as walking football (WF), has been developed in recent years. WF is a slower, low-impact adaptation of traditional football, specifically designed to encourage physical activity and social engagement among the older population. Research supports the effectiveness of walking football, along with other recreational sports such as soccer and basketball, in improving body composition [1, 2], cardiovascular health [3, 4], and muscle function [5].

Beyond these physiological benefits, WF enhances psychosocial well-being by fostering social interaction and boosting mood – factors, especially important for older adults, who may experience loneliness or social isolation [6, 7]. These combined physical and psychosocial benefits contribute to the growing popularity of WF among older populations [8].

Despite the physical and psychosocial benefits of football, it is also linked to a high incidence of musculoskeletal injuries. Injury rates can reach up to 8.1 per 1,000 hours of play, mainly affecting the lower limbs and occurring more frequently during matches [9]. Given these risks, it is worth exploring whether WF, with its rule modifications to reduce strain and injury risk, minimises injury in older adults.

Although the literature generally supports WF as a safe activity for older adults [5, 10–13], whether the sport’s rule modifications are sufficient to eliminate the risk of injury is not yet known. Some studies report injuries occurring in WF, which found an incidence of 5.3 injuries per 1,000 hours during training and 37.6 per 1,000 hours during matches, across several British teams [14]. However, these findings must be interpreted cautiously due to methodological limitations, including the use of self-reported data without medical confirmation and a heterogeneous sample, particularly in terms of team composition and age, which may have led to the underreporting or misclassification of injuries. Most of the existing literature has focused on short-term interventions [2, 4, 5, 10], with a notable lack of studies examining injury risk during regular, long-term participation. Moreover, research involving recreational or lower-tier competitive players is limited, despite their growing involvement in the sport and the need for more comprehensive data on injury incidence, severity and type [13].

Given that WF is predominantly practised by older adults, exploring specific risk factors related to this demographic is essential. The deterioration associated with ageing, particularly affecting the musculoskeletal system, may increase vulnerability to injury. Factors such as muscle function and metabolic health should be considered because metabolic risk factors have been linked to sarcopenia in individuals aged over 65 years [15]. Therefore, further empirical research is needed to accurately profile injury patterns and identify high-risk individuals in WF.

This study thus aimed to describe the epidemiology of injuries during training sessions and matches throughout a season in physically active older adults participating in WF. Specifically, it sought to characterise the circumstances of these injuries and determine their incidence and burden. Additionally, the study aimed to identify potential risk factors associated with muscle injury occurrence, comprising age, muscle function and blood biomarkers.

## MATERIALS AND METHODS

### Participants

Sixty-four recreational male WF players aged over 50 participated. They participated in a WF program that aimed to promote an active lifestyle and healthy ageing through an adapted and safe form of football. Participants engaged in training one to two days per week. The players had been participating in WF for a period ranging from 1 to 3 years. The recruitment of the players was conducted through meetings with participants of the WF program for older adults. During these meetings, the players were provided with all relevant information and given the opportunity to ask questions to the research team members. Those who agreed to participate were provided with the information sheet and signed the informed consent. All were medically cleared. They were subsequently scheduled to come to the laboratory to undergo all the necessary assessments and measurements. Training occurred twice weekly (1 h/session) from September to June, with occasional friendly matches and competitions. Training sessions were supervised by officially certified football coaches trained to work with diverse populations, including older adults (walking football). The study was approved by the Ethics Committee of the University of the Basque Country (EHU) (code GO 14/306-18) and followed the Declaration of Helsinki.

### Study design

A prospective follow-up was conducted throughout the competitive season with the 64 players to systematically record injuries and monitor individual exposure time during training sessions and official matches. At the beginning of the season, a baseline assessment was performed, which was completed by 43 participants. This initial evaluation included anthropometric measurements, blood samples, handgrip strength testing, a countermovement jump (CMJ) test and isokinetic strength assessments. The participants also completed sociodemographic and clinical questionnaires. They were instructed to maintain their usual dietary, medication and activity routines before the testing, while avoiding strenuous exercise on the day of assessment.

### Data collection

#### Anthropometry

Height (cm) and body mass (kg) were measured and body mass index (BMI; kg/m^2^).

#### Handgrip

Grip strength was measured using a Jamar handheld dynamometer (USA), expressed in kg. Participants sat comfortably and their elbow was flexed at 90°. Each hand was tested three times; the highest value was recorded [16].

#### Countermovement jump

Lower limb power was measured by performing a CMJ (Optojump platform, Microgate, Italy). Participants warmed up 10 minutes cycling at 60 rpm with 1 kg load [17]. They performed three CMJs with their hands on their waist, jumping maximally. The highest jump (cm) was used for analysis [18].

#### Isokinetic testing

Concentric quadriceps and hamstring strength were measured bilaterally using an isokinetic dynamometer (Humac Norm, USA) at 60°/s and 180°/s [19]. They performed three maximal reps per velocity, with 1-minute rest between velocities. The highest peak torque normalized to body weight (Nm/kg) was used.

#### Blood samples

Blood samples (5 mL) were collected in the morning after overnight fasting (7:00–11:00 a.m.). The assessed parameters were glycated haemoglobin (HbA1c; %), glucose (mg/dL), insulin (mcU/L), Homeostatic Model Assessment of Insulin Resistance (HOMA-IR), total cholesterol (mg/dL), high-density lipoprotein (HDL; mg/dL), low-density lipoprotein (LDL; mg/dL), atherogenic index – Castelli I (total cholesterol/HDL), triglycerides (TG; mg/dL), C-reactive protein (CRP; mg/dL), vitamin D (ng/mL), interleukin-6 (IL-6; pg/mL), creatine kinase (CK; U/L), lactate dehydrogenase (LDH; U/L), adiponectin and myostatin (ng/L).

#### Individual training and match exposure time

The individual exposure time of each player during training sessions and friendly or competitive matches was documented daily and recorded in minutes by a consistent member of the research team, who was responsible for monitoring all sessions to ensure standardised and accurate data collection. Training sessions, lasting approximately 1 hour and held twice weekly, primarily consisted of a brief warmup including light mobility exercises and low-intensity movements aimed at preparing the participants for physical activity. These were predominantly followed by small-sided games or short practice matches designed to replicate real-game scenarios and facilitate skill development in a controlled environment.

Before competitive matches, players performed a similar warmup routine intended to optimise their physical and physiological readiness. Match duration and format varied depending on the type of competition (friendly or official) and organisational factors, with games sometimes played as 5 v 5 or 6 v 6 and with different time limits. Matches were typically scheduled on weekends, based on demand or availability throughout the season, since no formal league was established.

#### Injury definitions, exposure and recording procedures

Injury was defined as any physical complaint preventing players from training or matches; illnesses were excluded. Players were considered injured until they returned to walking football. Time lost was recorded. Injuries were diagnosed and documented by an experienced physiotherapist of the research team, who attended all sessions and matches, ensuring consistent assessment following Fédération Internationale de Football Association (FIFA) guidelines [20]. Injuries were classified according to type, location, onset, mechanism and nature [20]. They were further grouped according to severity: minimal (1–3 days), mild (4–7 days), moderate (8–28 days), and severe (> 28 days) [20].

### Statistical analysis

Descriptive data are shown as frequencies, means ± standard deviation, medians and interquartile ranges (IQR). Injury incidence was defined as the number of new injuries occurring during a specific period relative to the total exposure time. It is expressed as the number of injuries per 1,000 player-hours, with 95% confidence intervals (CIs). Injury burden was calculated as the total number of days lost due to injuries per 1,000 player-hours of exposure, with 95% CI.

The sample size was relatively and conveniently in nature determined by the number of players attending the WF sessions and the number who provided informed consent for data collection [13, 14].

Potential risk factors (age, blood and strength parameters) were individually analysed for all muscle injuries. For that purpose, participants were divided into two age groups: over 50 (50–59 years old) and over 60 (≥ 60 years old). This is how age categories are typically organised in competitions. Likewise, quantitative blood and strength variables were categorized using the 30^th^ and 70^th^ percentiles as cutoff points: low (≤ 30^th^ percentile), middle (30^th^–70^th^ percentile), and high (≥ 70^th^ percentile). Injury incidence rate ratios (RR) (95% CI) of each age and percentile group were tested using z-statistics.

The possible relationships between risk factors and muscle injuries were assessed by the Cox proportional hazards model with a frailty extension, using the Survival package in R (version 4.2.2, R Foundation for Statistical Computing, Vienna, Austria) [21]. This model was applied, with age as a potential confounding factor, to calculate the adjusted hazard ratios (HR) of injury incidence with 95% CI. The model considers varying exposure times between players (measured as total hours of exposure), and the frailty term allows for correlation between observations from the same player [22]. Some players had no muscle injuries during the season, while others sustained one or more muscle injuries, and thus, had multiple observations. Kaplan Meier survival curves were used to illustrate the probability of remaining injury-free during a season.

The significance level was set at p < 0.05.

## RESULTS

A total of 2,946 player-sessions were recorded. On average, 47 ± 19 sessions were monitored per player, with a median of 42 (IQR: 37–62). The mean session attendance rate was 92.5%.

Table 1 shows the descriptive data of the participants and the values corresponding to the 30^th^ percentile (low), the 30^th^–70^th^ percentile range (middle) and the 70^th^ percentile (high) of the studied potential risk factors.

**TABLE 1:** Descriptive data of the variables. Means, standard deviations (SD) and values corresponding to the 30^th^ percentile (low), 30^th^–70^th^ percentile range (middle) and 70^th^ percentile (high) are reported.

Variable	Mean ± SD	Low	Middle	High
Age (years)	62.00 ± 7.00			
Height (cm)	172.86 ± 5.99			
Weight (kg)	82.87 ± 12.02			
HbA1c (%)	5.59 ± 0.51	< 5.3	5.3–5.6	> 5.6
Glucose (mg/dL)	97.65 ± 19.08	< 87.0	87.0–100.2	> 100.2
Insulin (mcU/mL)	7.72 ± 5.41	< 4.84	4.84–9.06	> 9.06
HOMA-IR	1.97 ± 1.77	< 1.13	1.13–2.26	> 2.26
TC (mg/dL)	197.76 ± 0.44	< 172.4	172.4–223.0	> 223
HDL (mg/dL)	56.33 ± 12.88	< 50	50–61	> 61
LDL (mg/dL)	120.45 ± 40.40	< 100.72	100.72–141.20	> 141.2
Atherogenic I-C	3.59 ± 0.79	< 3.2	3.2–3.9	> 3.9
TG (mg/dL)	108.20 ± 64.64	< 68.2	68.2–127.2	> 127.2
CRP (mg/dL)	0.22 ± 0.19	< 0.10	0.10–0.26	> 0.26
IL-6 (pg/mL)	3.29 ± 3.92	< 1.84	1.84–2.88	> 2.88
Vitamin D (ng/mL)	28.12 ± 8.82	< 23	23–30	> 30
Adiponectin (ng/L)	39.32 ± 12.94	< 32.77	32.77–47.75	> 47.75
Myostatin (ng/L)	2956.84 ± 770.43	< 2048	2048–2900	> 2900
CK (U/L)	236.02 ± 304.86	< 104	104–182	> 182
LDH (U/L)	360.43 ± 59.83	< 327	327–388	> 388
CMJ (cm)	18.66 ± 4.24	< 16	16–21	> 21
HG (kg)	44.56 ± 6.25	< 41.5	41.5–48	> 48
PT Q at 60°/s (Nm/kg)	2.08 ± 0.39	< 1.25	1.25–2.25	> 2.25
PT H at 60°/s (Nm/kg)	1.13 ± 0.23	< 1.13	1.03–1.25	> 1.25
PT Q at 180°/s (Nm/kg)	2.17 ± 0.52	< 1.95	1.95–2.33	> 2.33
PT H at 180°/s (Nm/kg)	1.25 ± 0.35	< 1.11	1.11–1.41	> 1.41

HbA1c: glycated haemoglobin; HOMA-IR: Homeostasis model assessment of insulin resistance; TC: Total cholesterol; HDL: highdensity lipoprotein; LDL: low-density lipoprotein; Atherogenic I-C: Atherogenic index – Castelli I; TG: triglycerides; CRP: C-reactive protein; IL-6: interleukin 6; CK: creatin kinase; LDH: lactate dehydrogenase; CMJ: countermovement jump: HG: handgrip; PT: peak torque; Q: quadriceps; H: hamstrings; °/s: degrees per second; Nm/kg: Newton-meters per kilogram.

Total, training and match exposure time are shown in Table 2. A total of 62 injuries occurred during the season: 80.6% sustained during training and 19.4% during matches. The most common were muscle injuries (64.5%), and injuries predominantly occurred during training (87.5%). Regarding the type of injuries, 64.5% were muscle strains, 16.1% ligament injuries and 11.3% tendon injuries. Of the latter, three were Achilles tendon ruptures, and the rest were tendinopathies. Of the total injuries, 29% affected the hamstring muscle and 25.8% the calf muscles.

**TABLE 2:** Descriptive data of participants and injuries.

Variable	Value
**Exposure time (hours)**	3148.8
Training	3007
Match	141.8

**Injuries [Number (%)]**	62 (100%)
Training	50 (80.6%)
Match	12 (19.4%)

**Muscle injuries [Number (%)]**	40 (100%)
Training	35 (87.5%)
Match	5 (12.5%)

**Type [Number (%)]**
Muscle injury	40 (64.5%)
Joint/ligament injury	10 (16.1%)
Tendon injury^*^	7 (11.3%)
Contusion	4 (6.4%)
Bone injury	1 (1.7%)

**Location [Number (%)]**
Upper limb	2 (3.2%)
Hip/groin	2 (3.2%)
Adductor muscles^#^	5 (8.1%)
Hamstring muscle^#^	18 (29.0%)
Quadriceps muscle^#^	1 (1.6%)
Knee	6 (9.7%)
Calf muscles^#^	16 (25.8%)
Achilles tendon^&^	3 (4.8%)
Ankle	8 (12.9%)
Foot	1 (1.6%)

* includes tendinopathy and tendon rupture;

# muscle strain;

& tendon rupture.

The injury incidence (95% CI) and burden (95% CI) of the injuries are reported in Table 3. Most injuries occurred in the thigh (38.3%), followed by the lower leg (30.6%). The most frequent injury mechanism was acute (90.3%), whereas chronic injuries accounted for a lower percentage (9.7%). Non-contact injuries were the most common (85.5%), compared to a lower proportion of contact injuries (14.5%). In terms of severity, 50% of the injuries were classified as moderate, 45.2% as severe and 4.8% as mild.

**TABLE 3:** Injury incidence and burden by location, mechanism, circumstance and severity.

Total	Number (%)	Injury Incidence (95% CI)	Injury Burden (95% CI)

	62 (100%)	19.69 (15.35–25.25)	892.40 (695.75–1144.63)
**Location**			
Head/neck	0 (0.0%)	0.00 (0.00–0.00)	0.00 (0.00–0.00)
Upper limb	2 (3.3%)	0.64 (0.16–2.54)	6.03 (1.51–24.12)
Trunk	0 (0.0%)	0.00 (0.00–0.00)	0.00 (0.00–0.00)
Lower limb	60 (96.7%)	19.05 (14.79–24.54)	880.65 (682.32–1136.64)
Thigh	23 (38.3%)	7.30 (4.85–10.99)	255.65 (169.889–384.72)
Knee	6 (9.7%)	1.91 (0.86–4.24)	66.69 (29.96–148.45)
Lower leg	19 (30.6%)	6.03 (3.85–9.46)	211.19 (134.71–331.10)
Ankle	8 (12.9%)	2.54 (1.27–5.08)	88.92 (44.47–177.81)

**Mechanism**			
Acute	56 (90.3%)	17.78 (13.67–23.10)	792.37 (609.8–1136.64)
Overuse	6 (9.7%)	1.91 (0.87–4.24)	100.03 (44.94–222.67)

**Circumstance**			
Contact	9 (14.5%)	2.85 (1.49–5.49)	93.36 (48.58–179.45)
Non-contact	53 (85.5%)	16.83 (12.86–22.03)	589.11 (450.07–771.12)

**Severity**			
Mild	3 (4.8%)	0.95 (0.31–2.95)	5.08 (1.64–15.76)
Moderate	31 (50.0%)	9.84 (6.92–14.00)	160.70 (113.01–228.50)
Severe	28 (45.2%)	8.89 (6.14–12.88)	726.63 (501.70–1052.39)

CI: confidence interval.

**FIG. 1:**
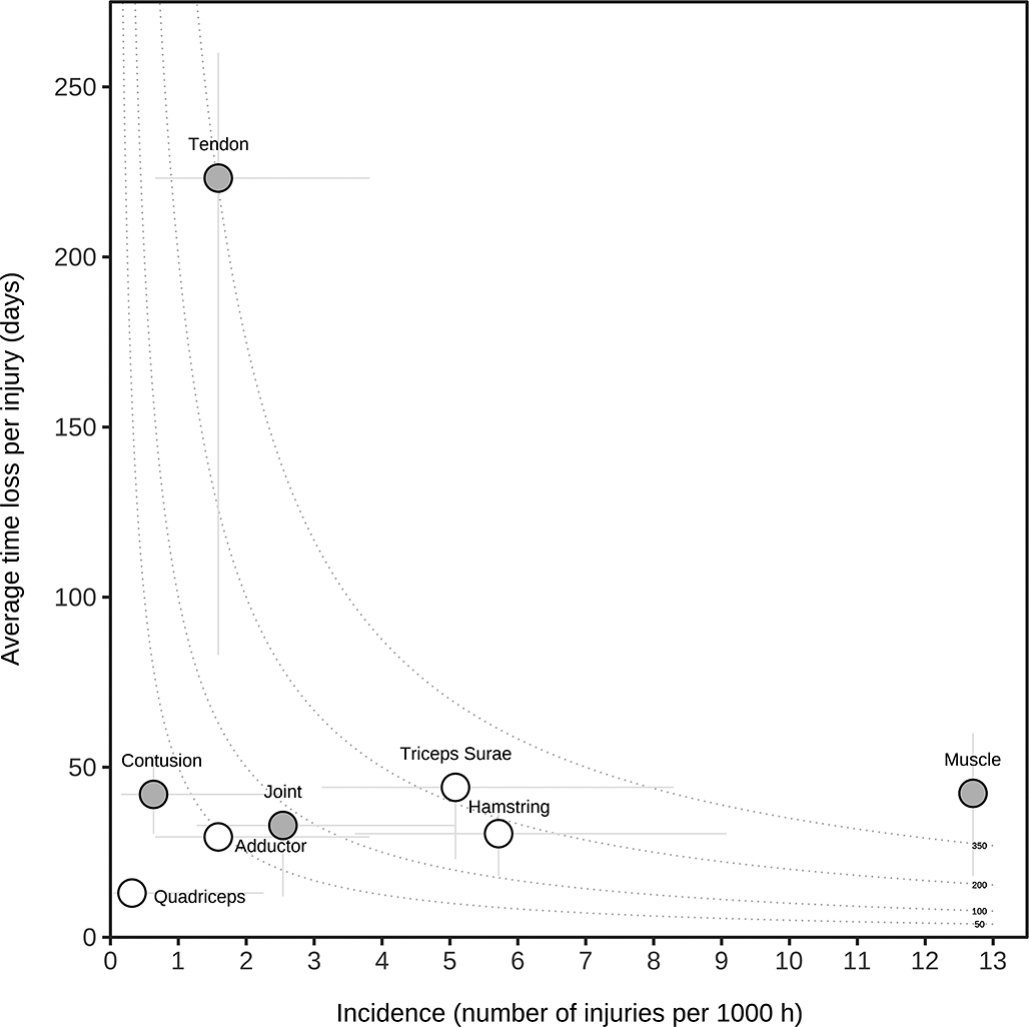
Risk matrix illustrating the injury burden of various injury types and specific muscle injuries. Data on injury types are represented as grey circles, and specific muscle injuries are represented as white circles. The curved dotted lines show points with equal burden. The vertical and horizontal error bars show 95% CIs.

Figure 1 shows the risk matrix of the injury burden and incidence of various injury types and specific muscle injuries. The high burden of tendon injuries (220 days absent/injury) and the high incidence of muscle injuries (12.9 injuries/1,000 hours) are noteworthy.

The analyses of the incidence of muscle injuries and blood and strength parameters are shown in Tables 4 and 5, respectively. The incidence of injuries in players with higher glucose levels was higher than in those with low glucose levels (p < 0.05). The incidence of muscle injuries was higher in players with low HDL values compared to those with high values (p < 0.05). Players with high atherogenity – Castelli Index I and high triglycerides had a higher risk of sustaining muscle injuries than players with middle or low values (p < 0.05).

Cox proportional hazards analysis revealed that individuals with low HDL had a higher risk of experiencing a muscle injury during the season compared to those with high HDL (HR of 4.39 [95% CI: 1.53–12.57], p = 0.006). The log-rank test confirmed that the survival distributions differed significantly between groups (p < 0.01). Players with high TG and high atherogenity – Castelli Index I had a higher risk of sustaining a muscle injury than players with middle and low values in those parameters (p < 0.05).

Age appeared to be associated with a higher risk of muscle injury, with players over 60 years old tending to have a greater risk compared to those aged over 50 years.

The injury incidence of older players (23.45 injuries per 1,000 hours [95% CI: 17.57–31.31]) was higher than that of younger players (14.32 injuries per 1,000 hours [95% CI: 8.90–23.03]; RR 1.68 [95% CI: 0.96–2.94]), statistically not significant. The burden of older players was higher (1,123.10 days absent per 1,000 hours [95% CI: 841.22–1499.41] than that of younger players (517.88 days absent per 1,000 hours [95% CI: 321.95–833.07]; RR: 2.23 [95% CI: 1.28–3.89]; p < 0.05). Notably, all tendon ruptures occurred in the over-60 age group.

**FIG. 2:**
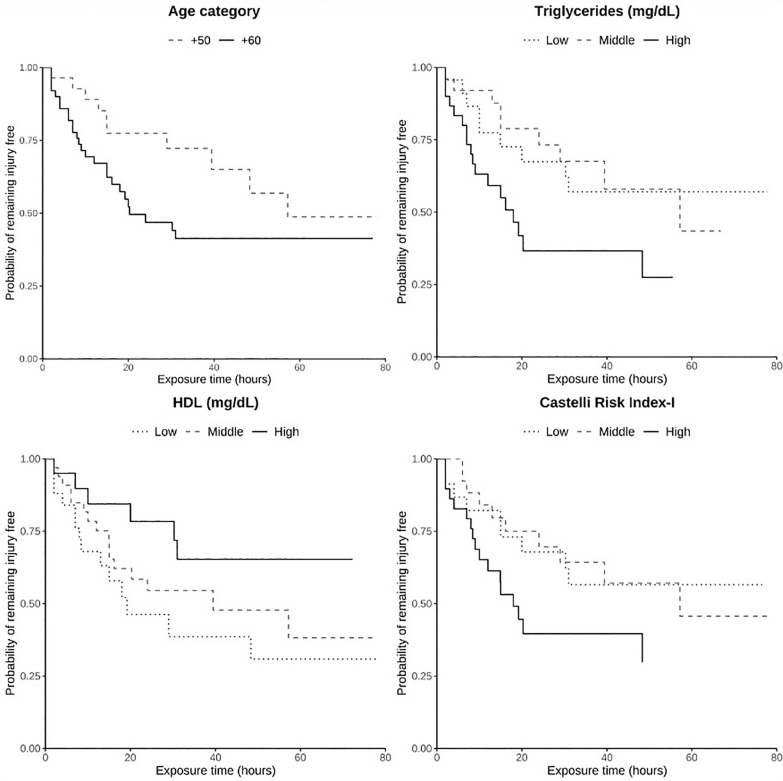
Kaplan–Meier survival curves showing the probability of remaining muscle injury–free during the season for the playing category and for those risk factors significantly related to muscle injuries.

In the specific group of muscle injuries, the incidence was higher in the older age group compared to the younger age group. The HR was 1.88 [95% CI: 0.93–3.82], although this difference did not reach statistical significance (p = 0.08; Table 4). Kaplan–Meier survival curves were used to estimate the time-to-injury distribution (Figure 2). Strength-related risk factors were not associated with the incidence of muscle injuries (Table 5).

**TABLE 4:** Analysis of blood sample–related risk factors for muscle injuries in walking football players. The incidence of the injuries in each percentile group and the comparison between groups are reported.

Variable	Group	Incidence (95% CI)	HR (95% CI)	p-value
Age group	Over 50	14.32 (8.90–23.03)	1.88 (0.93–3.82)	0.08
	Over 60	23.45 (17.57–31.31)		

HbA1c (%)	High	24.51 (13.88–45.56)		
	Middle	15.57 (9.22–26.30)	0.60 (0.27–1.37)	0.23
	Low	13.88 (8.06–23.91)	0.67 (0.29–1.56)	0.35

Glucose (mg/dL)	High	27.88 (16.51–47.07)^#^		
	Middle	14.50 (8.42–24.98)	0.59 (0.27–1.28)	0.18
	Low	11.83 (6.37–22.00)	0.49 (0.21–1.12)	0.09

Insulin (mcU/mL)	High	12.72 (6.36–25.43)		
	Middle	15.69 (9.46–26.03)	1.22 (0.51–2.94)	0.65
	Low	21.27 (12.59–35.91)	1.51 (0.63–3.63)	0.35

HOMA-IR	High	20.92 (12.14–36.02)		
	Middle	14.14 (8.03–24.90)	0.74 (0.34–1.65)	0.46
	Low	15.52 (8.81–27.33)	0.76 (0.35–1.68)	0.50

TC (mg/dL)	High	16.16 (8.95–29.18)		
	Middle	16.25 (9.62–27.44)	0.99 (0.45–2.20)	0.99
	Low	17.11 (9.72–90.14)	1.04 (0.46–2.39)	0.91

HDL (mg/dL)	High	8.87 (3.99–19.75)^#^		
	Middle	16.56 (10.43–27.03)	2.16 (0.84–5.58)	0.11
	Low	**24.96 (15.05–41.04)**	**4.39 (1.53–12.57)**	**0.006**

LDL (mg/dL)	High	14.26 (7.90–25.75)		
	Middle	13.83 (7.86–24.35)	0.94 (0.41–2.14)	0.89
	Low	23.16 (13.72–39.11)	1.34 (0.59–3.03)	0.49

AI – Castelli I	High	27.34 (17.23–43.40)^*^		
	Middle	**11.80 (6.35–21.23)**	**0.39 (0.17–0.87)**	**0.02**
	Low	**12.20 (6.35–23.45)**	**0.29 (0.11–0.76)**	**0.01**

TG (mg/dL)	High	31.00 (9.78–78.61)^*^		
	Middle	**11.10 (5.77–21.32)**	**0.37 (0.17–0.83)**	**0.02**
	Low	**10.98 (5.71–21.11)**	**0.38 (0.17–0.86)**	**0.02**

CRP (mg/dL)	High	18.59 (10.56–32.73)		
	Middle	11.90 (6.59–21.48)	0.79 (0.34–1.84)	0.59
	Low	20.18 (11.72–34.76)	1.16 (0.52–2.58)	0.71

IL-6 (pg/mL)	High	16.68 (9.47–29.37)		
	Middle	19.76 (12.11–32.26)	1.41 (0.64–3.11)	0.40
	Low	12.60 (6.56–24.22)	0.94 (0.39–2.26)	0.89

Vitamin D (ng/mL)	High	24.99 (15.07–41.45)		
	Middle	16.34 (9.28–28.78)	0.69 (0.32–1.51)	0.35
	Low	11.00 (5.92–20.45)	0.53 (0.22–1.29)	0.16

Adiponectin (ng/L)	High	16.82 (9.05–31.27)		
	Middle	15.77 (9.15–27.15)	0.95 (0.40–2.23)	0.91
	Low	16.98 (10.06–28.67)	1.09 (0.47–2.52)	0.83

Myostatin (ng/L)	High	15.93 (9.25–27.43)		
	Middle	12.14 (6.53–22.56)	0.91 (0.38–2.17)	0.83
	Low	23.19 (13.74–39.16)	1.53 (0.67–3.48)	0.31

CK (U/L)	High	19.75 (12.10–32.23)		
	Middle	9.27 (4.63–18.53)	0.46 (0.19–1.08)	0.08
	Low	22.81 (13.24–39.28)	1.06 (0.51–2.23)	0.87

LDH (U/L)	High	20.86 (12.58–34.60)		
	Middle	12.71 (7.53–24.46)	0.72 (0.34–1.50)	0.37
	Low	18.93 (9.47–37.85)	0.96 (0.40–2.67)	0.93

CI: confidence interval; HR: hazard ratio; HbA1c: glycated haemoglobin; HOMA–IR: Homeostasis Model Assessment of Insulin Resistance; TC: total cholesterol; HDL: high-density lipoprotein; LDL: low-density lipoprotein; AI: atherogenic index; TG: triglycerides; CRP: C-reactive protein; IL-6: interleukin 6; CK: creatin kinase; LDH: lactate dehydrogenase; CMJ: countermovement jump: HG: handgrip; PT: peak torque; Q: quadriceps; H: hamstrings

# p < 0.05 vs. low;

* p < 0.05 vs. middle and low.

**TABLE 5:** Analysis of strength-related risk factors for muscle injuries in walking football players. The incidence of the injuries in each percentile group and the comparison between groups are reported.

Variable	Group	Incidence (%95 CI)	HR (%95 CI)	p-value
CMJ (cm)	High	13.78 (7.83–24.27)		
	Middle	17.04 (9.90–29.36)	0.95 (0.40–2.30)	0.92
	Low	18.74 (10.38–33.84)	1.05 (0.41–2.62)	0.93

HG (kg)	High	19.31 (11.44–32.61)		
	Middle	11.70 (6.48–21.13)	0.63 (0.28–1.40)	0.26
	Low	20.74 (11.78–36.53)	0.87 (0.37–2.03)	0.75

PT Q at 60°/s (Nm/kg)	High	9.58 (4.57–20.09)		
	Middle	9.15 (4.76–17.58)	0.67 (0.29–1.52)	0.34
	Low	11.14 (4.63–26.75)	0.76 (0.29–1.52)	0.57

PT H at 60°/s (Nm/kg)	High	7.47 (3.11–17.96)		
	Middle	10.55 (5.68–19.61)	0.76 (0.34–1.68)	0.49
	Low	10.97 (4.93–24.42)	0.71 (0.28–1.80)	0.36

PT Q at 180°/s (Nm/kg)	High	6.46 (2.43–17.22)		
	Middle	12.07 (6.69–21.80)	1.35 (0.57–3.16)	0.82
	Low	8.68 (3.61–20.86)	0.88 (0.31–2.53)	0.49

PT H at 180°/s (Nm/kg)	High	7.11 (2.96–17.09)		
	Middle	11.81 (6.15–22.70)	1.18 (0.49–2.85)	0.75
	Low	9.35 (4.20–20.80)	0.85 (0.31–2.33)	0.70

CI: confidence interval; HR: hazard ratio; CMJ: countermovement jump: HG: handgrip; PT: peak torque; Q: quadriceps; H: hamstrings; °/s: degrees per second; Nm/kg: Newton-meters per kilogram

## DISCUSSION

WF has been recognised as a valuable intervention to enhance cardiovascular, metabolic and psychosocial health in older adults. However, despite promising outcomes, evidence remains lacking regarding the potential risks associated with its practice. This is particularly relevant given that WF targets an older population, which is inherently more susceptible to injury. We thus aimed to describe and report the incidence and burden of injuries sustained during a WF season. Furthermore, we sought to identify risk factors associated with injury occurrence to determine the most vulnerable participants.

The main findings revealed a notable number of injuries among participants, specifically muscle injuries. The majority occurred in the posterior compartment of the leg muscles and were predominantly acute and non-contact in nature. While muscle injuries were not associated with muscle function, significant associations were observed with certain blood parameters. Age also emerged as a relevant factor, as older participants experienced both a higher incidence and, more importantly, a greater burden of injuries. These results provide novel insights into the injury profile of older WF players and contribute important evidence to guide safer implementation of WF in this population.

WF is a relatively new sport and, consequently, limited publications have addressed its risks, particularly regarding injury epidemiology. Throughout the entire season, a total of 62 injuries were recorded in our investigation. In another study, eight injuries (strains, sprains, and contusions) were reported during a 12-week WF program involving 31 men (mean age: 64 years), with sessions held three times per week (60 minutes each) [10]. The reported musculoskeletal injury incidence was 9.3 injuries per 1,000 player-sessions, which is lower than the incidence found in our study. Moreover, only two injury events (joint pain and a hamstring tear) were reported among 25 prostate cancer patients participating in a 16-week WF program (90-minute sessions once per week) [5]. The lower injury incidence reported in these previous studies may be related to the limited duration of these interventions and the fact that they were conducted within research settings, where participants were likely subject to closer supervision and more controlled conditions than in real-world practice. Additionally, neither study reported a detailed analysis of the characteristics or mechanisms of each injury. Similarly, lower figures have been reported elsewhere; for example, a recent study conducted during a WF championship matches documented 34 cases requiring medical attention but eight time-loss injuries, corresponding to injury rates of 40.0 [95% CI: 27.0–54.1] and 9.4 [95% CI: 3.5–16.5] per 1,000 hours, respectively [13].

Injuries were evaluated among WF players from seven clubs over 4 months. Although their total exposure time was almost double that of our study (6,364 *vs.* 3,148 hours), only 45 injuries were reported compared to the 62 recorded in our cohort [14]. Consequently, the overall incidence rate in our study was considerably higher (19.7 *vs.* 7.1 injuries per 1,000 hours). This discrepancy could be attributed to methodological differences. In that study [14], injury and exposure data were collected through individual player reporting or by team coaches or captains, which may have introduced an underreporting bias. Additionally, their sample included both male and female participants, although the gender distribution was not specified. Considering that women in professional football typically experience an estimated 30–40% fewer injuries than men, this factor may help explain the lower incidence reported [23]. Age differences may also contribute to the observed discrepancies. Whereas participants in the study [14] study ranged from 40 to 70 years old, our cohort had a mean age of 62.7 years (50.8–77.5). Age has been identified as a potential risk factor for injury in veteran football. In a study applying the same methodological framework as ours (FIFA Consensus Statement, 2006) [20], 14 injuries were reported in the 50–59-year-old group (14.4 injuries/1,000 hours [95% CI: 7.3–21.5]), which is comparable to our results in the same age group. Notably, the largest injury incidence was observed in the over-60 age group, which is consistent with the findings of our study.

These comparisons suggest that both methodological differences and participant age are important factors that must be considered when interpreting injury incidence in WF. Importantly, age was not only a variable to consider in interpretation but also a factor that influenced the results. Older participants experienced a higher incidence and, more notably, a greater burden of injuries. The most severe injuries were sustained by those in the oldest age group. This highlights the need for targeted prevention strategies even within the context of an adapted sport such as WF.

Other factors likely also contributed to the high injury incidence and burden in our study. Most injuries occurred in non-contact situations, supporting WF as a suitable activity for older adults due to its rule modifications aimed at reducing physical contact and injury risk. However, the injury incidence was around 20 per 1,000 hours, with a burden of 892 days absent/1,000 hours – higher than figures reported in younger professional [9] and amateur players [24], and with a greater severity than in professional soccer [23].

This disparity may stem from age differences, methodological factors and lower training volume in WF (approximately 2 hours/week) compared to professionals, who benefit from extensive training, medical support and structured conditioning programmes. WF sessions focused mostly on playing, with little complementary physical preparation. This suggests that incorporating additional training components aimed at enhancing physical fitness could be a feasible strategy to reduce injury risk and potentially improve performance, although further research is required to confirm this.

Muscle injuries predominated, especially hamstrings. This is consistent with findings in elite [23] and veteran footballers [25] and racewalkers [26], where the gait mechanics mimic the movement characteristics of WF.

The calf muscles were the second most affected site (25.8%). Additionally, we recorded three Achilles tendon ruptures. Although the incidence of this injury was low, it was notably higher than in previous WF studies [14, 25]. Importantly, these injuries have high clinical relevance due to their substantial burden, impacting both return to activity and daily functioning, which are particularly relevant in older adults.

Taken together, these observations highlight the posterior compartment of the lower leg as a key area of concern for injury in WF. This is likely due to sport-specific demands requiring rapid walking while avoiding running, which places high loads on the soleus, gastrocnemius and plantaris muscle complex [27, 28].

Another contributing factor in this older adult population is the degenerative changes of the Achilles tendon associated with ageing [29]. Tendon degeneration is linked to increased rupture risk [30], making it a significant concern in master athletes (ACSM, 2010) [31].

Prevention strategies should thus focus on these injuries. To reduce the risk of Achilles tendon rupture, ACSM (2010) [31] guidelines for master athletes recommend muscle strength, balance, and flexibility of the gastrocnemius and soleus complex, along with the kinetic chain of the lower extremity and a focus on functional balance and eccentric training.

Although biomechanical and technical factors contribute to these injuries, the involvement of deeper underlying molecular and metabolic factors cannot be ruled out. Our biochemical analyses revealed significant associations, highlighting the multifactorial nature of these injuries.

In this context, to our knowledge, this is the first study specifically aimed at elucidating injury risk factors within the domain of WF. To achieve this objective, we examined a range of variables, including blood biomarkers and muscular strength. Muscle functionality was not associated with the incidence of muscle injuries. Conversely, a higher incidence of muscle injuries and a higher risk for sustaining muscle injuries was observed among players with a dyslipidaemic profile, particularly those presenting low HDL cholesterol, elevated triglyceride levels and a high atherogenic index. Additionally, we found that elevated glucose levels (statistically significant), along with non-significant increases in HbA1c and decreased insulin levels, were also related to a higher incidence of muscle injuries. All these findings align with the characteristic features of metabolic syndrome.

According to current evidence, no studies have specifically examined a distinct lipid and glucose profile in relation to muscle injuries. Exploring this potential association in greater depth would thus be valuable for future research, especially considering that the existing literature has established a link between metabolic syndrome – which encompasses altered lipid profiles, hyperglycaemia and insulin resistance – and sarcopenia [32]. For instance, elevated levels of the atherosclerotic index of plasma, defined as the logarithm of the ratio of triglycerides to HDL cholesterol, have been associated with an increased risk of sarcopenia [33]. This relationship appears to be mediated, at least in part, by mitochondrial function [34]. Moreover, recent studies have demonstrated a direct connection between lipid profiles and mitochondrial health; specifically, higher circulating levels of HDL-C, ApoA-I and larger-sized HDL particles are independently associated with enhanced skeletal muscle mitochondrial function [35].

Focusing specifically on tendon injuries, our study identified three severe cases that represented a considerable burden. Although we cannot confirm a direct link between tendon injuries and metabolic alterations in our cohort, it is noteworthy that a similar dyslipidaemic profile has been reported in other contexts. Specifically, it has been associated with Achilles tendinopathy in runners with metabolic syndrome [36], with altered tendon structure and pain [37], with Achilles tendon rupture [38], and with rotator cuff ruptures and re-ruptures [39].

Moreover, the link between dyslipidaemia and tendon pathology appears to be mediated, at least in part, by insulin resistance syndrome [40]. Therefore, further studies examining these risk factors in relation to tendon injuries would be of interest.

### Methodological considerations

This study has several limitations. The sample size was relatively small, limiting generalisability despite high participation from the provincial league. Only male players were included, excluding potentially different injury patterns in women. Additionally, incomplete baseline data restricted risk factor analysis, particularly for Achilles tendon injuries, which, although severe, are rare and statistically challenging to study. Future research should involve larger, more diverse cohorts to overcome these limitations.

## CONCLUSIONS

This study provides novel insights into the epidemiology and risk factors of injuries in older adults participating in WF. We found an elevated overall injury incidence and burden, with muscle injuries— particularly affecting the posterior compartment of the leg—emerging as the most common. Importantly, older age was associated with a tendency toward an elevated injury incidence and a significantly greater severity of injuries, underscoring the vulnerability of this population despite the adapted nature of the sport.

Further research is required to confirm whether incorporating additional training components aimed at enhancing physical fitness could be a feasible strategy to reduce injury risk and potentially improve performance, as WF sessions focused mostly on gameplay.

Our findings also suggest a potential role of metabolic factors, including dyslipidaemia and impaired glucose regulation, in predisposing participants to muscle injuries. While these associations require further confirmation, they highlight the importance of considering metabolic contributors to injury risk in WF. In addition, the occurrence of Achilles tendon ruptures, although infrequent, represents a clinically significant concern given their impact on function and recovery in older players.

In summary, while WF offers valuable health benefits for older adults, injury prevention strategies must be prioritised to ensure safe participation. Larger, more diverse cohorts and prospective studies are needed to confirm these findings, further clarify underlying risk factors, and optimise evidence-based recommendations for injury prevention in WF.

## Data Availability

The data that support the findings of this study are available on request from the corresponding author, SMG.
